# The evolutionary ecology of bird–ant interactions: a pervasive but under-studied connection

**DOI:** 10.1098/rspb.2023.2023

**Published:** 2024-01-03

**Authors:** Jesús M. Avilés

**Affiliations:** ^1^ Departamento de Ecología Funcional y Evolutiva, EEZA-CSIC, Almería E-04120, Spain; ^2^ Unidad Asociada (CSIC-UNEX): Ecología en el Antropoceno, Badajoz E-06006, Spain

**Keywords:** antagonism, ant–bird interactions, commensalism, competition, eco system function, mutualism

## Abstract

Birds and ants are among the most ubiquitous taxa co-occurring in terrestrial ecosystems, but how they mutually interact is almost unknown. Here, the main features of this neglected interaction are synthetized in a systematic literature review. Interaction with ants has been recorded in 1122 bird species (11.2% of extant species) belonging to 131 families widely distributed across the globe and the avian phylogeny. On the other hand, 47 genus of ants (14.4% of extant genus) belonging to eight subfamilies interact with birds. Interactions include competition, antagonism (either ant–bird mutual predation or parasitism) and living together commensally or mutualistically. Competition (48.9%) and antagonism (36.1%) were the most common reported interactions. The potential for engaging in commensalism and competition with ants has a phylogenetic structure in birds and was present in the birds' ancestor. Interaction is better studied in the tropics, in where the network is less dense and more nested than in temperate or arid biomes. This review demonstrates that ant–bird interactions are a pervasive phenomenon across ecological domains, playing a key role in ecosystem function. Future studies need to combine sensible experimentation within anthropogenic disturbance gradients in order to achieve a better understanding of this interaction.

## 1. Introduction

Interactions play a fundamental role in moulding the structure and function of communities, and constitute the backbone of ecosystems. Communities result from the combination of biological entities and processes, so that to understand their functioning it is essential to know the species that compose them, but also how they interact [1,2], as this shapes the dynamics and stability of biodiversity [3].

Birds and ants are among the most ubiquitous and diverse animal groups playing key functional roles in most terrestrial ecosystems on earth [4,5]. Ants and birds are enormously abundant and species-rich taxa in the same communities [6], and therefore likely to encounter each other or share key resources. Indeed, some studies have shown that both birds and ants can act as predators in bird–ant interactions [7,8]. In addition, empirical work has reported a maintenance ‘anting behaviour’ during which birds rub ants on their feathers and skin [9]. Ants and birds might compete for food [10], or for nesting sites (either tree cavities or unenclosed nests) where ants can forage on other nest-dwelling invertebrates or on remains of bird feathers, droppings, etc. [11], but also benefit from a warm microclimate to raise their own broods under suitable thermal conditions [12,13]. In the New World and African tropics, many bird species associate with massive swarm-raiding of army ants capturing arthropods flushed out by the ants [14,15]. In addition, ants may protect birds against predation, as inferred from a high daily survival rate of avian nests located in trees with ants [16]. However, most studies so far have only considered one species of bird and one ant species, or where performed in one single biome, which is an oversimplification considering the large number of species of birds and ants coexisting in terrestrial ecosystems. Moreover, ant–bird interactions are often neglected due to the extraordinary diversity of ants, which makes their taxonomical identification a challenging task [17]. Thus, understanding about the interactions between ants and birds is surprisingly scarce and fragmentary, and the existing literature has not yet been synthesized.

The aim here is to focus the study of ant–bird interactions within the framework of evolutionary ecology. To achieve this, I conduct a survey to understand the main features of this invertebrate–vertebrate interaction. Specifically, I describe the diversity of birds and ants engaged in interactions. In addition, I qualify the ecological relevance of interactions detected in the survey in relation to the research approached used to investigate them. Moreover, I explore the geographical, taxonomic and phylogenetic extent of ant–bird interactions, and assess their importance in the different biomes of the earth. Also, aiming to characterize the structure of bipartite ant–bird interactions, I compare the topology of bird–ant networks in different biomes and among types of interaction using network analyses. The emerging patterns are discussed in light of ecosystem services provided by ant–bird interactions and potential bias of the literature search.

## 2. Material and methods

### (a) The database

To identify studies dealing with ant–bird interactions, a systematic literature review was performed, following the PRISMA recommendations [18] (electronic supplementary material, figure S1), using the Web of Knowledge database by combining the following keywords. Search 1: ‘ant' and ‘bird’ and ‘interaction’; search 2: ‘ant’ and ‘bird’ and ‘diet’; search 3: ‘ant’ and ‘bird’ and ‘predation’; and search 4: ‘ant’ and ‘bird’ and ‘habitat selection’; search 5: ‘ant’ and ‘bird’ and ‘mutualism’; search 6: ‘ant’ and ‘bird’ and ‘commensalism’; search 7: ‘ant’ and ‘bird’ and ‘parasitism’; search 8: ‘ant’ and ‘bird’ and ‘competition’; and search 9: ‘anting’ and ‘birds’. The literature search returned 1112 studies, of which 314 were removed because they were duplicates. In addition, 436 studies were excluded after reading their abstract and title, and verifying that they did not deal with ant–bird interactions (electronic supplementary material, figure S1). Hence, 362 articles were selected for full-text screening, of which 152 were excluded because they do not provide evidence of ant–bird interaction and/or reported an interaction already reported for a locality (electronic supplementary material, figure S1). Six additional articles were added after reviewing the references of the selected articles. Summing up, 216 articles contributed with interactions to the database (electronic supplementary material, figure S1), although a given study can include hundreds of interactions (electronic supplementary material, appendix S1).

For each study, all the interactions were recorded following the mechanistic definition of interaction by Abrams [19]. That is, ants and birds interact when there is a contact between them that entails an immediate effect because they are a consumer or resource, or they provide a service to each other. Hence, studies reporting spatial relationships between numbers of ants and birds were disregarded, as co-occurrence in broad habitat scales does not provide true evidence of interaction [20]. Interactions were categorized based on the actions performed by or properties of each effector as:

(1) *Competition* (i.e. interaction in which each of ants and birds affects the other by consumption of a commonly used set of resources) (electronic supplementary material, table S1). Two types of study provide support for competition: (i) studies reporting changes in bird foraging activity and/or habitat selection, and in insect prey abundance in response to ant removal; and (ii) studies reporting the use of a common resource (either food or refuge) by birds and ants. Therefore, in my consideration of competition, it is assumed that the use of common resources results in competition under low resource availability [21]. As most studies reporting competition were observational (see Results), it could not be deciphered if competition occurs by interference, by exploitation or was apparent [22], except for a few experimental studies that will be briefly described on individually.
(2) *Antagonism* (i.e. ants or birds benefit from the other species by consuming it totally or partially, potentially leading to the death of the prey) (electronic supplementary material, table S1). In this context, predation is an antagonistic interaction where the consumer kills the resource, and parasitism would be an antagonistic interaction in where the consumer does not kill the resource. Four types of study revealed antagonism: (i) studies reporting ant consumption by birds; (ii) studies reporting the predation of avian eggs or nestlings by ants; (iii) studies reporting active anting by birds; and (iv) studies measuring army ants' failure in prey capture in relation to bird attendance.
(3) *Commensalism* (i.e. either ants or birds gain benefit while the other species neither is benefited nor harmed) (electronic supplementary material, table S1). Three types of study suggested commensalism: (i) studies of birds following army ants in tropical forests; (ii) studies documenting the attraction of ants by avian nests where ants benefit by accessing to nutrients or favourable thermal environments at apparent no costs for birds; and (iii) studies reporting bird nest site choice and reproductive outcome in relation to ant presence.
(4) *Mutualism* (i.e. win–win interaction where both ants and birds benefit) (electronic supplementary material, table S1). Two types of study provide some support for mutualism: (i) studies reporting coexistence in avian nests suggesting benefits for ants and birds; and (ii) studies reporting passive anting.

**Figure 1.**
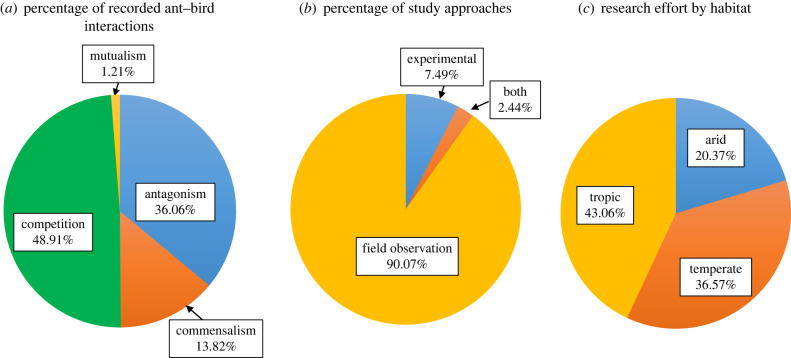
Summary of published studies describing ant–bird interactions. (*a*) Percentage of different interactions. (*b*) Different study approaches. The ‘both’ category involves studies that combined observations with experiments. (*c*) Percentage of studies in temperate, arid and tropical biomes.

This categorization of interactions is open to alternative interpretations because knowing the nature of interactions may require experimentation [23,24]. However, pragmatically it cannot be adopted an experimental-based approach to synthetize current knowledge on ant–bird interactions because most of evidence is based on observations (figure 1; electronic supplementary material, appendix S1). Hence, I opted to consider observational studies in the synthesis, but critically qualifying the strength of inference based on them (electronic supplementary material, table S1).

From each study source, I also retrieved the bird and ant taxonomic identities engaged in the interaction, the country and the biome where the interaction took place. Species was considered the taxonomic identity for birds. However, genus was considered as a lower taxonomic level for ants because species-level information for ants was missing for a large number of studies. The generic level is a useful taxonomic level at which to consider patterns of ant diversity [25]. Biomes were initially classified based on the 14 world terrestrial ecoregions defined by Olson *et al*. [26], but subsequently grouped into three main categories of tropical, temperate and arid following Gómez *et al*. [27], to ensure enough samples in each type of biome to make reliable inference (electronic supplementary material, appendix S1).

Interactions between same ant and bird taxonomic identities in different biomes were considered as independent raw cases in the database. Studies about consumption or transport of invertebrates, seeds or fruits, but in which ants and birds were not taxonomically identified, were not considered as they do not provide taxonomic inference.

### (b) Phylogenetic signal

Aiming to explore the tendency of related taxa to resemble each other from the way they interact, the phylogenetic signal of interactions was estimated using the phylo.d function in the CAPER package in R [28]. This approach is based on calculation of the statistic *D* [29], the value of which ranges continuously from 0 to 1. *D* values close to 1 would indicate that the presence of interaction is random regarding the phylogeny, which would support the idea of multiple origins. Instead, a *D* value close to 0 would indicate that the presence of interactions evolved under a Brownian model, which would support the idea of just a few origins followed by subsequent diversification.

### (c) Ancestral character reconstruction

The ancestral state of interactions on the avian phylogeny was reconstructed using the R function ‘reroothingMethod’ in the PHYTOOLS package [30]. This approach allows estimating the marginal ancestral state for each internal node of the tree using likelihood and comparing the performance of various models of evolution. Specifically, it was contrasted the ‘equal rates’ model (ER hereafter), which assumes that interaction presence is lost or acquired at a similar rate over time; and the ‘all rates different’ model (ARD hereafter), which allows for differences in the rate of gain and loss of an interaction. The Akaike information criterion was used to compare the models and select the best one, considering as support to a choice a difference of 4 or greater between models [31].

### (d) Network analyses

Bipartite interaction network analyses were used to explore for generalizable patterns about how ant–bird interactions are assembled in different biomes. First, one interaction matrix for each main category of biome (i.e. tropical, temperate and arid) lumped across all realms was compiled (electronic supplementary material, appendix S3). Secondly, a matrix for each type of interaction was compiled in order to analyse if the network of the different interactions differed (electronic supplementary material, appendix S3). Bipartite graphs were created based on bird species versus ant genus matrices using the R package 'bipartite' v. 2.17 [32], and their topology was described using metrics calculated with the functions ‘networklevel’ and ‘grouplevel’. The selected metrics at the network level were:

(1) *Connectance* (*C*), which quantifies the global density of interactions in a network as the proportion of possible links actually observed in a web [33].
(2) *Complementary specialization* (*H*_2_′), which ranges from 0 to 1 and describes the degree of niche divergence (i.e. niche complementarity) between bird species and ant genus in the interaction networks and, hence, the degree of complementary specialization in the network [34]. When species are specialized on different association partners (i.e. high niche differentiation) *H*_2_′ network increases.
(3) *Weighted nestedness*, or more specifically ‘weighted interaction nestedness estimator’, which quantifies nestedness (i.e. the degree to which the interactions of less-connected nodes are a subset of those of more connected nodes [35]), with 1 indicating perfected nestedness.

Moreover, two metrics at the group-level were used:

(4) *Generality*, as the weighted average number of interacting ant genus per bird species.
(5) *Redundancy*, as the weighted average number of interacting avian species per ant genus.

Network metrics are ecologically uninformative if either are driven by constraints in web dimensions or are a consequence of the lognormal distribution of taxa abundances [33,36]. To partly alleviate this issue, null models based on the Patefield algorithm [37] were built with the function ‘nullmodel’ in Bipartite [38] to assess whether network metrics differed from random expectation. To test for differences from randomness, observed values for the different metrics were compared with those obtained from 1000 permutations of randomized networks.

## 3. Results

### (a) Number and identity of interacting birds

The interaction of birds with ants is a widely recognized phenomenon, being reported in 1122 bird species included in 131 families (*n* = 2388 interactions; electronic supplementary material, table S2). Hence, interactions were observed in about 11.2% of bird species and 67.2% of families considered in AVONET [39]. Considering their functional traits [39], there exists huge variation in the primary lifestyle of avian species interacting with ants (electronic supplementary material, appendix S2). There are reports from insessorial (*n* = 633) to terrestrial (*n* = 243), aerial (*n* = 64), aquatic (*n* = 1) or generalist bird species (*n* = 129). Moreover, there are species living in all kind of habitats, from strictly forest species to those inhabiting deserts or rocky habitats (electronic supplementary material, appendix S2). A large proportion of bird species interacting with ants belong to the order Passeriformes (77.4%, electronic supplementary material, appendix S2). This percentage is higher than the percentage of Passeriformes species in AVONET (59.7% of species, $\chi_{1}^{2}=128.87$, *p* < 0.0001), suggesting that species in the order Passerifomes would be prone to interact with ants, or that interactions with ants were more detectable and/or studied in this order.

### (b) Number and identity of interacting ants

In total, 47 genera of ants included in eight subfamilies were reported to interact with birds (electronic supplementary material, table S3). The most abundant subfamilies in the database are Myrmicinae, Formicinae and Dorylinae, which accounted for 35.6%, 25.7% and 16.2% of the interactions, respectively (electronic supplementary material, table S3). Ants included in the subfamilies Myrmicinae and Formicinae are the most diverse group of ants interacting with birds, with 19 and 9 genera represented, respectively (electronic supplementary material, table S3). Considering that the diversity of extant ant species is distributed among 327 genera and 17 subfamilies worldwide [17], I have reported evidence of interaction with birds in 14.4% and 47.0% of ant extant genera and subfamilies, respectively.

### (c) Types of interactions between ants and birds

In figure 1*a* the relative importance of the different interactions is summarized. Ants and birds interact in a number of ways, including competing for resources, antagonistically as predator/prey or parasitically, and living together commensally or mutualistically (figure 1*a*).

#### (i) Competition

Competition was by far the most frequent interaction (48.91% of all interactions; figure 1*a*), the bulk of interactions coming from observational studies reporting ants and birds eating on common food resources (electronic supplementary material, appendix S1). A handful of experimental studies reported a positive effect of ant removal on the abundance of invertebrates on trees, and on the foraging activity of birds, providing support for exploitation competition [10,40,41].

Moreover, experimental evidence provides support for interference competition. An ant exclusion experiment of high-density super-colonies of yellow crazy ant *Anoplolepis gracilipes* showed that ants directly interfere with fruit handling by birds [42]. Experiments also revealed that hummingbirds avoid flowers with ants [43].

#### (ii) Antagonism

Antagonism was the second most frequent interaction (36.1% of interactions; figure 1*a*). Most evidence came from studies reporting ant consumption by birds (627 out of 861 (72.8%); electronic supplementary material, appendix S1). In whole, 513 avian species ate ants distributed in all habitats and biomes (electronic supplementary material, appendix S1), which would contrast with the widespread belief that the presence of distasteful and potentially toxic formic acid in ants would lead many species of birds to avoid them [44].

Many bird species also engage in active anting (19.4% of antagonistic interactions; electronic supplementary material, appendix S1), whereby they squeeze ants and rub them on their plumage, presumably in an attempt to take advantage of the insecticidal properties of formic acid.

Moreover, a number of studies also recorded egg or nestling predation by ants [45,46], or identified disturbance effects by ants on avian nestling development [8,47]. Overall, the survey provides support for a predatory role of ants in 47 avian species (electronic supplementary material, appendix S1), and demonstrates that both ants and birds can play a dual role as either prey or predator.

Parasitism was very rarely reported (0.29% of antagonistic interactions; electronic supplementary material, appendix S1). Evidence came from a study showing that *Eciton burchellii* success in capturing prey increased after the removal of ant-follower birds [48].

#### (iii) Commensalism

Birds and ants can also live together adopting apparently commensal interactions (13.82% of interactions; figure 1*a*). Most evidence came from observations of birds following army ant raids in tropical forests [15,49], and does not estimate costs of bird attendance for ants (see however [48]) or birds (see however [50]). Commensalism may also fit with the occasional observation of ants being attracted to avian nests where they feed on food resources, and the observation of nestlings being reared with success in nests with an egg-producing colony of ants [11,51]. Maziarz and co-workers [13,52,53] also showed high occurrence between wood warblers *Phylloscopus sibilatrix* and ants in the primeval Białowieża Forest, and that warbler activity in their nests created warm microclimate suitable for fast development of ant larvae. Active nests of birds, warmed up by the owners, attracted ants raising their own broods, and so they could be an important resource of warm nesting sites for ants in cool environments.

Commensalism also fits with the observation of parasite control by ants at no apparent cost to birds. Brown *et al*. [54] discovered that *Crematogaster lineolata* and *Formica* spp. ants preyed on swallow bugs *Oeciacus vicarius*, reducing their numbers on active swallow nests without any effect on the reproductive success of cliff swallows.

Finally, several bird species show a preference to locate their nests in acacia trees with *Pseudomyrmex* spp. ants in the Neotropic. By protecting the acacia tree, the ants may inadvertently reduce the risk of avian nest predation, whereas the ants and the tree are not affected by bird presence [55]. Experimental work in which bird nests were located in trees with and without ants corroborated that the presence of ants may enhance avian reproductive success by reducing nest predation[16].

#### (iv) Mutualism

Reports of mutualism are anecdotal (1.21% of interactions; figure 1*a*), and deserve experimental confirmation. Conner & Lucid [56] reported that carpenter ants *Camponotus* spp. coexisted in cavities with nestling common flickers *Colaptes auratus* without any detrimental effects to the nestlings. They suggested that ants forming a colony would benefit by excavating the tunnels and chambers of their nests in the rotten heartwood of trees previously opened by common flickers, whereas common flickers would benefit by eating some ant individuals while excavating [56]. Mutualism may also fit with observations of ant species predating on avian ectoparasites [57,58]. Birds might benefit from parasite control exerted by ants, whereas ants might benefit by accessing to food resources produced by birds or from suitable thermal conditions for raising their broods within bird nests, two possibilities deserving experimental confirmation. Finally, there are a few reports of passive anting, in which birds intentionally lie on an ants' nest and allow them to crawl all over their bodies [59]. The ants release acids that presumably act as an insecticide to remove parasites from the bird's feathers.

### (d) Phylogenetic distribution and evolutionary origin of interactions

**Figure 2.**
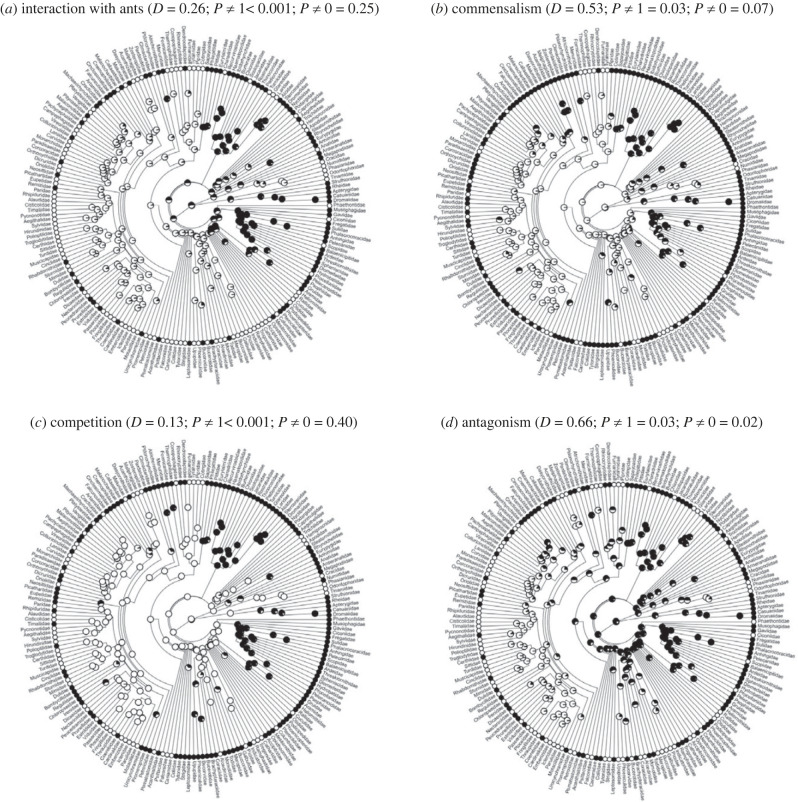
Phylogenetic relationships of avian families interacting with ants: (*a*) any interaction; (*b*) commensalism; (*c*) competition; and (*d*) antagonism. White and black dots at the tips indicate presence or absence of interactions, respectively. Dots at the nodes represent proportional maximum-likelihood support for the presence (white) or absence (black) of interaction from the ancestral state reconstruction. The phylogenetic signal was calculated with the parameter *D* [29], and the phylogenetic hypothesis is based on Jetz *et al*. [60].

The interaction with ants is not restricted to a distinct part of the avian phylogeny (figure 2). Commensalism, competition and antagonism with ants (but not mutualism) that only occur in 14 avian families (electronic supplementary material, figure S1) are present in several distant avian families (figure 2).

The study of phylogenetic signal revealed that there is a strong phylogenetic structure for the presence of interactions with ants, and for commensalism and competition, because *D* departs significantly from random expectation, but does not differ from expectation under a Brownian model of evolution (figure 2). However, antagonism was not phylogenetically conserved (figure 2). The phylogenetic structure for the presence of interaction with ants is not due to a clumped distribution of research effort among bird families (electronic supplementary material, appendix S5).

Ancestral reconstructions revealed that the probability that the bird ancestor would have interacted with ants could not be established (likelihood at the root: 0.45, figure 2*a*). However, birds' ancestor was probably involved in commensal and competitive interactions with ants (likelihoods at the root: 0.95 and 0.99, respectively; figure 2*b,c*). By contrast, antagonism with ants probably evolved at a later stage of avian evolution (likelihood at the root: 0.81 for the absence of antagonism, figure 2*d*).

On the other hand, the presence of interactions with birds in the ant phylogeny is scarce, occurs in distant genera and showed no phylogenetic structure (electronic supplementary material, figure S2).

### (e) Geographical and ecological distribution of ant–bird interactions

**Figure 3.**
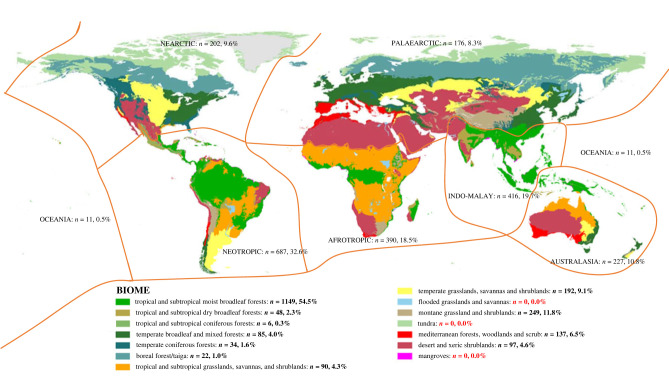
Global geographical location of interactions. Fourteen global biomes within eight biogeographic realms (source: Olson *et al*. [61]). Number of interactions and percentage of the whole in each biome and realm are shown in the legend, and within the limits of the realm.

Ants and birds interact in all the terrestrial biogeographical realms except in the rock and ice of the Poles, where ants are absent [25] (figure 3). Therefore, ant–bird interactions occur all over the world rather than being restricted to particular biomes or regions. Nevertheless, the proportion of interactions in the Neotropic, the Indo-Malay and the Afrotropic realms are comparatively larger than in others (figure 3).

Interactions occur in a wide variety of habitats, being absent only in tundra, flooded grasslands, savannas and mangroves (figure 3), and are more frequently observed in tropical biomes (59.2%) than in temperate (31.5%) or arid (9.3%) ones. It is worth noting that the study of interactions in the tropics has a long tradition. Indeed, the 43.06% of the 216 studies considered were performed in tropical biomes (figure 1*c*).

### (f) Network topology by biome

**Figure 4.**
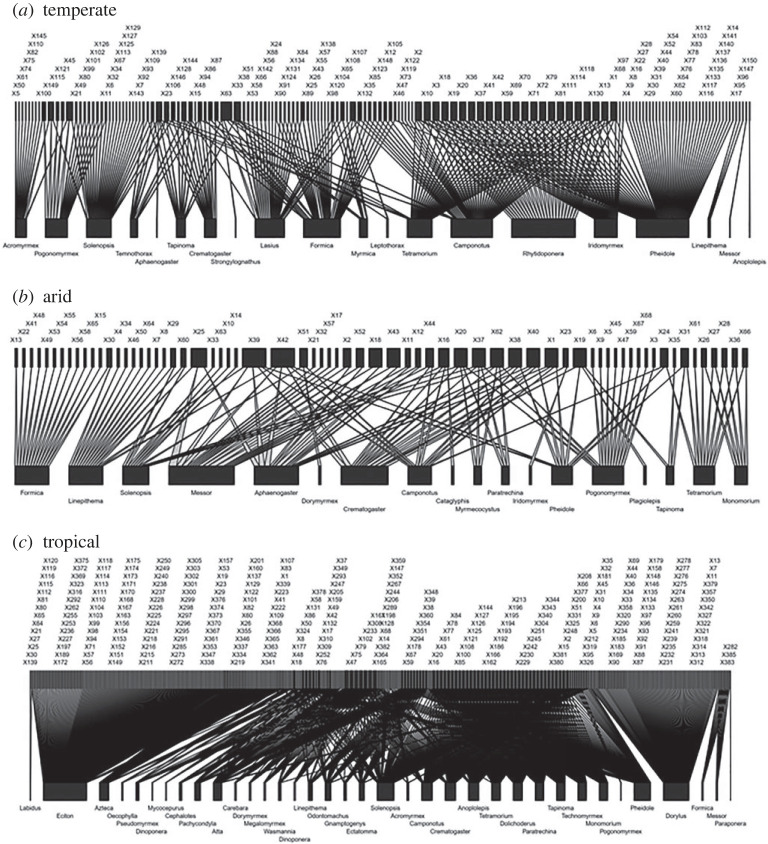
Weighted bipartite networks representing interactions in (*a*) temperate, (*b*) arid and (*c*) tropical biomes. Each graph represents the proportion of interactions between avian species (upper bars) and ant genera (lower bars). The width of the rectangle represents the total number of interactions, and the widths of the connecting lines represent the number of interactions observed for that link. Codes for bird species are described on the electronic supplementary material, appendix S3.

Network metrics reveal differences in how ant–bird communities are assembled between temperate and tropical biomes: connectance (*C*) is higher, and there are more bird species supporting single or few ant interactors (i.e. high complementary specialization (*H*_2_′), table 1) in temperate than in tropical networks. The values of these two metrics differ from random expectation in the two biomes, but not in the arid habitat (table 1). Moreover, nestedness in tropical biomes is significantly higher than predicted by random expectation, and than that in temperate and arid biomes (table 1 and figure 4).

**Table 1.** Network metrics calculated for bird–ant interactions in temperate, arid and tropical biomes, and interaction type. Signs between brackets indicate whether observed metrics were significantly higher (+), lower (−) or not different (ns) from random expectation based on the Patefield algorithm [37] built with the function nullmodel in bipartite.

		biome			
**metric**	level	temperate	arid	tropical	
connectance (*C*)	network	0.11 (−)	0.11	0.07 (+)	
specialization degree (*H*_2_′)	network	0.27 (+)	0.19	0.04 (−)	
weighted nestedness	network	0.46	0.43 (+)	0.69 (+)	
number of bird species	taxonomic group	150	68	385	
number of ant genus	taxonomic group	20	18	36	
generality (birds)	taxonomic group	3.09	2.88	7.22	
redundancy (ants)	taxonomic group	27.08	10.47	61.70 (+)	

		interaction type			
metric	level	antagonism	commensalism	competition	mutualism
connectance (*C*)	network	0.06	0.06 (−)	0.10 (+)	0.20^a^
specialization degree (*H*_2_′)	network	0.17	0.59 (+)	0.07 (−)	0.00^a^
weighted nestedness	network	0.39	0.86 (+)	0.43	—
number of bird species	taxonomic group	91	258	274	9
number of ant genus	taxonomic group	23	16	38	7
generality (birds)	taxonomic group	2.52	1.26	7.64	2.14^a^
redundancy (ants)	taxonomic group	10.61	103.82	50.51 (+)	4^a^

^a^Comparison with null model is not possible due to simplicity of network.

Regarding group metrics, only redundancy in tropical biomes is high and higher than expected by chance (table 1), showing that a given ant genus is prone to interact with more avian species.

### (g) Network topology by interaction type

**Figure 5.**
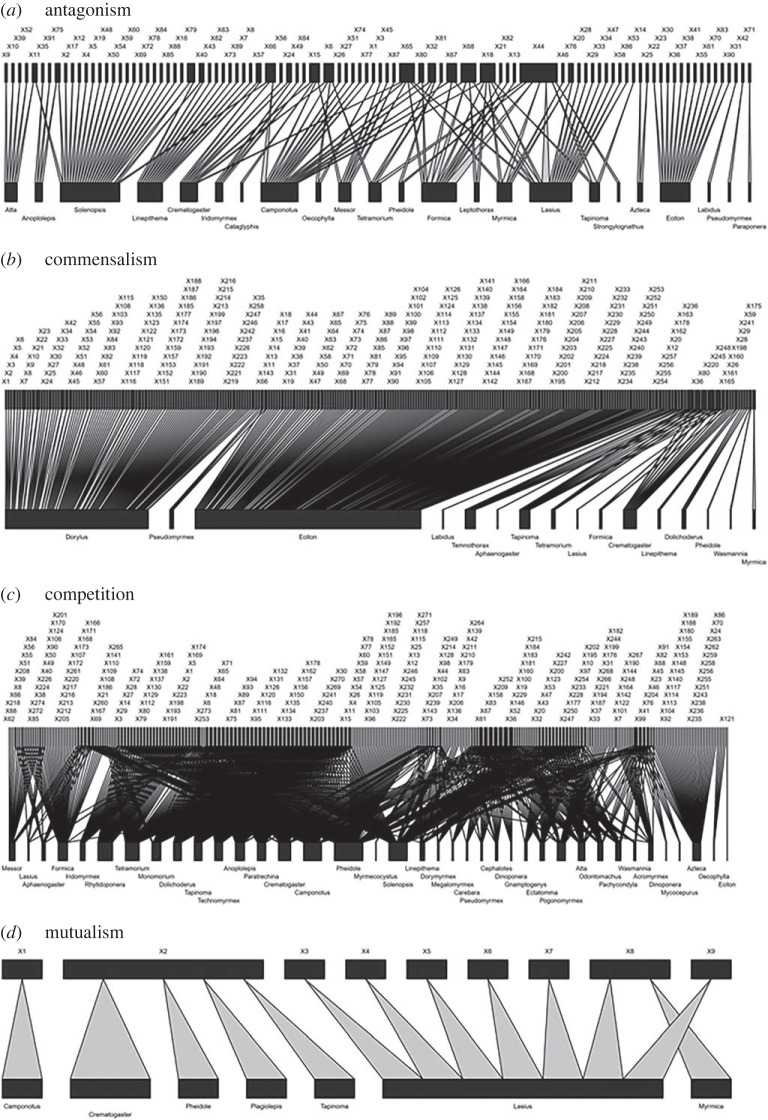
Weighted bipartite networks representing (*a*) antagonistic, (*b*) commensal, (*c*) competitive and (*d*) mutualistic interactions. Each graph represents the proportion of interactions between avian species (upper bars) and ant genus (lower bars). The width of the rectangle represents the total number of interactions, and the widths of the connecting lines represent the number of interactions observed for that link. Codes for bird species are described in the electronic supplementary material, appendix S3.

The interactions differ in how they are assembled (figure 5). The antagonistic and mutualistic networks do not differ from random expectation (table 1). However, the competition network showed significantly higher levels of connectance and lower complementary specialization than the network of commensal interactions (*H*_2_′; table 1). Moreover, nestedness is significantly higher than expected by chance and high in the network of commensalism (table 1).

Group metrics show that redundancy in the competition network is high and significant, suggesting that a given ant genus is prone to compete with many avian species (table 1 and figure 5).

## 4. Discussion

Ants and birds interact in most biomes and regions on Earth (figure 3). Interactions involve a highly diverse group of ant and bird species with a wide phylogenetic distribution and differing greatly in their lifestyle. In the case of birds, the high prevalence of interactions with ants is similar to that reported for other well-studied interactions. For example, 1089 bird species have been identified as pollinators [62]. It is worth noting that the collated dataset included only bird species with published evidence of interaction with ants. However, the study of ant–bird interaction was rarely the main target of research of these studies. Thus, the true extent of interactions with ants will be more widespread in birds than is documented here. In the same vein, this review only considered studies in which ants could be identified to the genus level. Hence, it is likely that interactions with birds are much more frequent in ants than is reported here, and with the data at hand, it is demonstrated that they take place in many disparate lineages of ants.

Ant–bird interactions range from antagonism (predation interactions in both directions and parasitism) to competition, commensalism or mutualism (figure 1*a*). The survey revealed that the relative importance of interactions is variable, which may reflect the difficulties for identifying interactions through observational studies. Almost 90% of interactions in the database are based on field observations (figure 1*b*). Identifying interactions often requires measuring costs and benefits of the interaction for the partners while manipulating their presence. Experiments might be logistically unpractical and unrealizable whenever the research questions involved a wide geographical and ecological perspective [63]. Therefore, observational studies might be useful at the early stages of research for identifying ant–bird co-occurrence, guiding further research in some interactions. In addition, observations can feasibly serve in the aim of detecting antagonism by predation [7], but it is undeniable they have a limited potential for identifying interactions into the antagonism–commensalism–mutualism continuum. Consequently, antagonistic interactions based on predation are probably overrepresented in this study.

Ant–bird interactions do not show a high degree of specialization, probably because the association between the two taxa is mostly temporary due to the high mobility of birds relative to ants. This would have hampered the evolution of more specialized interactions in which ants frequently engage with sessile organisms [64]. In this sense, the most likely durable interactions between birds and ants, in avian cavity nests, or those taking place in the nests of some raptors, corvids or storks, may constitute an ideal and tractable system for the study of specialized interactions.

Ant–bird interactions are more frequent and diverse in tropical biomes than in temperate and arid ones. This pattern is similar to that reported for other ant interactions. For instance, the proportion of Lycaenidae butterflies interacting with ants decreases from the tropics towards the north [65]. More generally, many biotic interactions are more frequent in the tropics than in temperate regions [66]. The importance of the tropical biome for ant–bird interactions could be due to the high degree of speciation of birds and ants [67]. However, the tropics have long attracted the attention of ecologists of interactions, and particularly ant–bird interactions (figure 1*c*). Hence, there is likely to be bias in the data gathered and it is necessary to perform multi-replicated studies in the different biomes to qualify the importance of ant–bird interactions at a global scale.

### (a) Phylogenetic distribution and origin of interactions

There was a strong phylogenetic structure for the presence of interactions with ants among birds, but not so for the presence of interactions with birds among ants. It could be argued that ants diversified before birds, and could have lost the shared traits favouring their interactions with birds, which might have resulted in a lower phylogenetic signal for ants. Ants probably originated in the Late Jurassic or Early Cretaceous, around 100 Ma, whereas the subfamilies Myrmecinae and Formicinae, to which most ant genera interacting with birds belong, probably appeared during the Late Cretaceous (90 Ma [68]). Meanwhile, Passeriformes (the most interacting order of birds) evolved around 50 Ma [69]. However, simulation-based studies have shown that the correspondence between evolutionary rate and phylogenetic signal is complex and that our ability to make inference about evolutionary processes when dealing with low phylogenetic signals is very limited [70]. The low number of ant genera with information about interactions with birds rather than differences in the rate of evolution would be the most feasible explanation for the low phylogenetic signal in ants.

The potential to interact commensally or compete with ants has a strong phylogenetic structure and was held by the ancestors of birds. The fact that several extant phylogenetic distant reptilian taxa had been reported to have commensal [71] and competitive [72] interactions with ants might suggest that even the reptilian ancestor of birds already had that proneness to interact with ants. However, although the birds ‘ancestor engaged in commensalism and competition with ants, many avian families currently do not, which suggests either that some bird families may have loss that capacity while radiating because selection led then to exploit niches where they do not coincide with ants, or that these families were less studied. By contrast, antagonism with ants would be a more labile trait appearing later in the evolution of birds.

It is worth stressing that ant–bird interaction has been analysed as a trait that can be selected upon. Interaction, however, might be contingent on abiotic and biotic factors (e.g. [58]), and the coincidence of birds and ants in time and place, which are aspects that are not entirely dependent on genetic inheritance. Hence, the potential of the comparative analyses to infer neat evolutionary patterns along the avian phylogeny should be expected to be low. Despite this, I found a phylogenetic structure for the interactions that birds have with ants, which would suggest that these interactions can be the target of selection and conserved phylogenetically.

### (b) Topology of interactions

The biome influences the way in which ant–bird networks are assembled. The tropical network is composed of more avian or bird taxa, but it is less connected and more nested than the network in temperate biomes due to the high occurrence of interactions between ants of the genera *Dorylus* and *Eciton* and the many bird species that attend their raids (figure 4). The high occurrence of this interaction determines that the network of commensal interactions will be less connected than the network of competitive interactions (figure 5). Nested networks are particularly vulnerable to species invasions [73], hence ant–bird interactions in the tropics would be particularly sensitive to these impacts.

### (c) Why is it important to study ant–bird interactions?

Ant–bird interactions play key ecosystem functions that can be inferred from two sources of evidence. The first are ‘natural experiments’ in which ecological communities are affected by the arrival of non-native species. For instance, the introduced yellow crazy ant*,* which has rapidly expanded its range through the islands of the Indian Ocean, engages in interference competitively with endemic birds, potentially eroding key ecological services they provide, such as seed dispersal [42]. Moreover, the arrival of a bird predator, the brown tree snake *Boiga irregularis*, relaxed predation control of birds on ants with potential effects on herbivory in the Mariana Islands [74].

Exclusion experiments also provide support for a key role of ant–bird interactions on ecosystems. Birds and ants can predate on invertebrates and seeds, and their experimental removal revealed changes in top-down control over the arthropod communities [75,76] and seed dispersal [77], with potential consequences on herbivory and plant recruitment. Furthermore, a handful of fully factorial designs allow identifying which are the individual effects of birds and ants on ecosystems, but also if these are independent, or contingent upon each other [78]. The action of ants and birds on seed predation in the Chilean Andes would be additive, and dependent on the type of seed, affecting plant recruitment [79]. Also, Denmead *et al*. [80] excluded ant and flying vertebrate (birds and bats) access to oil palms in Sumatra, and found effects on arthropod communities but overall weak effects on ecosystem functions (herbivory, predation, decomposition and pollination). A similar experiment in cacao plantations in Indonesia, however, revealed that birds and ants exhibited strong independent top-down control on herbivores and predators, affecting cacao yield, but no interactive effects among bird and ant exclusions [6]. So far, a single study has reported non-additive effects of birds and ants while studying the individual and combined effects of birds (chickadees, nuthatches, warblers) and ants *Formica podzolica* on arthropods residing in pine *Pinus ponderosa* canopies [78]. Summing up, a number of studies have reported a possible role of ant–bird interactions in ecosystem function, and experiments have allowed identification of which are the individual and combined effects of birds and ants [81]. However, there are remarkable differences between communities and regions in the predatory role of ants and birds that make it necessary to continue collecting information in order to establish generalizations in this regard.

### (d) Weakness of the used approach and prospects

This literature review has several weaknesses worth mentioning that may affect the strength of the conclusions. First, data gathered are most based on observational or even anecdotal reports, which may have resulted in overestimation of direct interactions of antagonism and competition; and it was difficult to locate some interactions within the antagonistic–parasitism continuum. Second, the outcome of interactions depended on the presence of other species (e.g. [82]) or environmental conditions (e.g. [58]). Qualifying the role of contingency on interactions is far out of the reach of this review given that interaction records come predominantly from a temporally static perspective. Nonetheless, ant–bird interactions provide a tractable system for studying interactions within environmental gradients, and for addressing the role of contingency (see e.g. [60]). Third, in this review I have pragmatically adopted a categorical classification of interactions. However, there is a growing consensus that their outcome falls somewhere along continuums between purely positive and negative effects for the partners [83]. Hence, patterns reported here may represent snapshots of different stages of a continuous gradient of interactions. Future studies should endeavour to identify the interaction costs for both parties, and study the role of biotic and abiotic contingence on these interactions. The integration of the study of costs within anthropogenic gradients emerges as an enormously promising research area to explore.

## Data Availability

The data are provided in electronic supplementary material [84].
